# Supplementary material for: Gleditsia triacanthos Galactomannans in Gluten-Free Formulation: Batter Rheology and Bread Quality
Source: Foods. 2023 Feb 9;12(4):756. doi: 10.3390/foods12040756 (PMC9956313; doi:10.3390/foods12040756)
Supplement: Supplementary file 1 [file foods-12-00756-s001.zip › foods-2172003-supplementary.pdf]

foods

MDPI

Supplementary Material

Tables

Table S1. Rheological parameters of gluten-free batters.

Sample	Storage modulus (Pa)	Loss modulus (Pa)	tan δ	Complex viscosity (Pa.s)
Control	5710±183 a	1630±70 a	0.286±0.005 a	945±31 a
Guar 0.5%	30,600±3277 b	8520±613 b	0.279±0.018 a	5055±526 b
Guar 1.25%	619,250±154812 d	169,475±42,368 d	0.272±0.068 a	102,150±25,537 d
Guar-Xanthan 0.5%	11,5425±17783 c	28,675±3712 c	0.249±0.008 a	18,925±2863 c
Guar-Xanthan 1.25%	661,250±165,312 de	189,750±47,437 de	0.283±0.071 a	109,425±27,356 de

Different letters within a column are significantly different ($p < 0.05$).

Table S2. Textural parameters of gluten-free batters.

Sample	Unfermented batter		Fermented batter	
	Firmness (g)	Young's modulus (Pa)	Firmness (g)	Young's modulus (Pa)
Control	23±4 a	2129±694 a	21±2 a	5923±1692 a
Guar 0.5%	50±1 b	6883±718 b	26±3 ab	5768±1859 a
Guar 1.25%	138±13 d	8839±1228 c	30±5 b	14,551±880 c
Guar-Xanthan 0.5%	95±8 c	7542±902 b	46±4 c	10,474±3022 b
Guar-Xanthan 1.25%	220±8 e	13,149±708 d	80±13 d	7965±2135 ab

Different letters within a column are significantly different ($p < 0.05$).

Table S3. Gluten-free bread specific volume and crumb structure.

Sample	Cell area (%)	Cell/mm^2	Cell size (mm)	SBV (cm^3/g)
Control	48.5±0.3 a	0.65±0.02 c	0.75±0.02 a	1.99±0.30 ab
Guar 0.5%	50.6±0.3 c	0.47±0.02 b	1.08±0.05 bc	2.18±0.12 bc
Guar 1.25%	50.3±0.2 c	0.44±0.02 b	1.16±0.05 c	2.30±0.28 c
Guar-Xanthan 0.5%	50.0±0.2 bc	0.31±0.01 a	1.58±0.02 d	2.13±0.18 abc
Guar-Xanthan 1.25%	48.8±1.0 ab	0.48±0.04 b	0.99±0.02 bc	1.93±0.16 a

Different letters within a column are significantly different ($p < 0.05$).

Table S4. Gluten-free bread textural parameters.

Sample	Firmness (N)			Staling rate	Chewiness (ad)		
	Day 0	Day 1	Day 3	(N/day)	Day 0	Day 1	Day 3
Control	5.6±1.2 d	16.1±2.0 d	42.7±8.9 c	12.51	6.7±1.3 d	14.2±1.5 d	23.6±5.8 c
Guar 0.5%	3.7±0.5 b	8.1±0.9 b	18.0±4.5 b	4.81	4.3±0.5 b	7.5±1.1 b	9.5±2.2 b
Guar 1.25%	2.7±0.5 a	6.2±0.9 a	11.6±2.2 a	2.94	3.1±0.6 a	5.9±0.8 a	6.0±1.2 a
Guar-Xanthan 0.5%	4.1±0.7 bc	8.7±1.7 bc	18.4±3.4 b	4.78	5.0±0.8 bc	8.4±1.8 bc	11.2±1.9 b
Guar-Xanthan 1.25%	4.8±0.7 c	9.5±0.7 c	18.7±1.4 b	4.65	5.8±0.8 c	9.4±0.7 c	11.9±1.2 b

Different letters within a column are significantly different ($p < 0.05$).

Table S5. Crumb water activity.

Sample	a_w		
	Day 0	Day 1	Day 3
Control	0.977±0.001 a	0.976±0.001 a	0.967±0.003 a
Guar 0.5%	0.979±0.001 a	0.976±0.001 a	0.972±0.001 b
Guar 1.25%	0.978±0.001 a	0.977±0.002 a	0.972±0.002 b
Guar-Xanthan 0.5%	0.978±0.001 a	0.976±0.002 a	0.970±0.002 ab
Guar-Xanthan 1.25%	0.978±0.001 a	0.977±0.002 a	0.972±0.001 b

Different letters within a column are significantly different ($p < 0.05$).

Table S6. Gluten-free bread crust color.

Sample	L*	a*	b*	ΔE*
Control	72.2±4.6 a	2.5±0.4 c	20.3±3.0 bc	-
Guar 0.5%	76.1±1.3 b	3.4±0.7 d	25.2±2.1 d	6.33
Guar 1.25%	80.3±1.0 c	1.9±0.1 ab	19.4±1.1 b	8.17
Guar-Xanthan 0.5%	80.5±0.5 c	2.4±0.4 bc	22.5±1.3 c	8.59
Guar-Xanthan 1.25%	80.3±0.9 c	1.5±0.2 a	15.6±1.2 a	9.42

Different letters within a column are significantly different ($p < 0.05$).

Figures

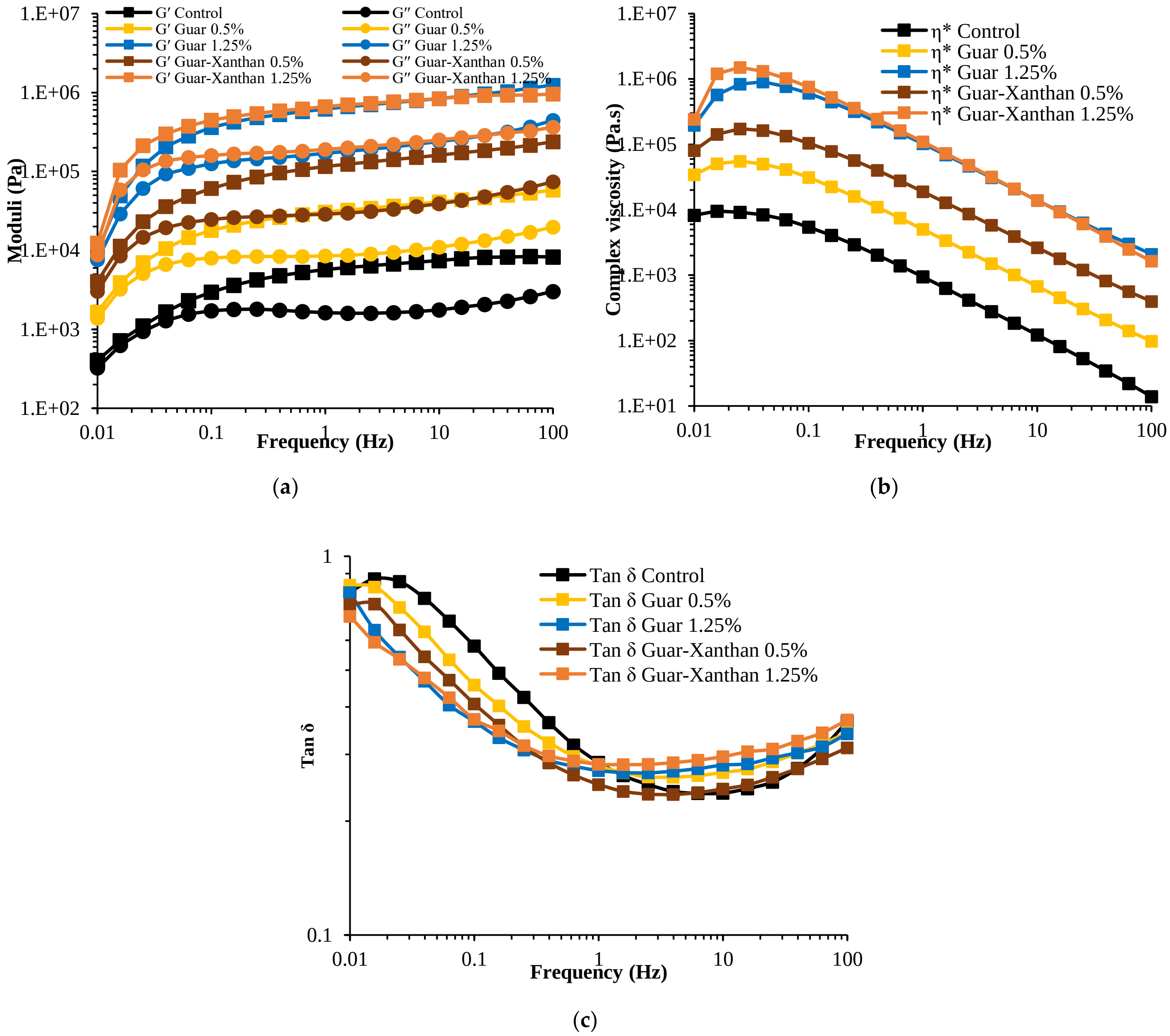

Figure S1. Rheograms for all studied samples. (a) Storage and loss moduli, (b) Complex viscosity, (c) tan δ

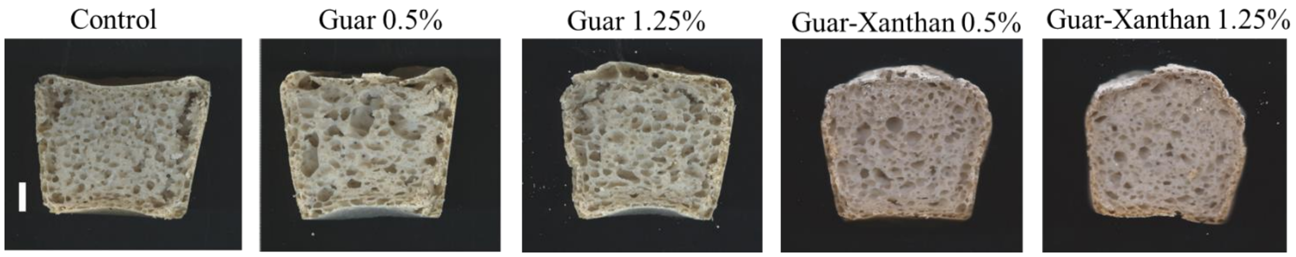

Figure S2. Representative images of gluten-free bread. Bar: 1 cm.
